# Epidemiology and Genetic Diversity of PCV2 Reveals That PCV2e Is an Emerging Genotype in Southern China: A Preliminary Study

**DOI:** 10.3390/v14040724

**Published:** 2022-03-30

**Authors:** Quanming Xu, Yongyi Zhang, Wen Sun, Hong Chen, Dewen Zhu, Chang Lu, Yuanyuan Yin, Kul Raj Rai, Ji-Long Chen, Ye Chen

**Affiliations:** 1 Key Laboratory of Animal Pathogen Infection and Immunology of Fujian Province, College of Animal Sciences, Fujian Agriculture and Forestry University, Fuzhou 350002, China; xqmfafu@163.com (Q.X.); zyy3693@163.com (Y.Z.); sunwen1905649784@163.com (W.S.); chenhoo0766@163.com (H.C.); zhudw0809@163.com (D.Z.); lc1447391649@163.com (C.L.); yinyuanyuan117@163.com (Y.Y.); kulrajrai701@gmail.com (K.R.R.); chenjl@im.ac.cn (J.-L.C.); 2 Fujian Police College, Fuzhou 350007, China

**Keywords:** PCV2e, co-infection, genetic diversity, mutation

## Abstract

Porcine circovirus-associated disease (PCVAD), caused by porcine circovirus type 2 (PCV2), has ravaged the pig industry, causing huge economic loss. At present, PCV2b and PCV2d are highly prevalent genotypes worldwide, while in China, in addition to PCV2b and PCV2d, a newly emerged PCV2e genotype detected in the Fujian province has attracted attention, indicating that PCV2 genotypes in China are more abundant. A preliminary study was conducted to better understand the genetic diversity and prevalence of PCV2 genotypes in southern China. We collected 79 random lung samples from pigs with respiratory signs, from 2018 to 2021. We found a PCV2-positivity rate of 29.1%, and frequent co-infections of PCV2 with PCV3, *Streptococcus suis* (*S. suis*), and other porcine pathogens. All PCV2-positive samples were sequenced and subjected to whole-genome analysis. Phylogenetic analysis, based on the PCV2 ORF2 gene and complete genomes, found that PCV2 strains identified in this study belonged to genotypes PCV2a (1), PCV2b (6), PCV2d (10), and PCV2e (6). Importantly, PCV2e was identified for the first time in some provinces, including Guangdong and Jiangxi. Additionally, we found two positively selected sites in the ORF2 region, located on the previously reported antigenic epitopes. Moreover, codon 63, one of the positively selected sites, has different types of amino acids in different genotypes. In conclusion, this study shows that PCV2e is an emerging genotype circulating in southern China, which warrants urgent, specific surveillance to aid the development of prevention and control strategies in China.

## 1. Introduction

Porcine circovirus (PCV) is a single-stranded, circular DNA virus belonging to the *Circovirus* genus of the *Circoviridae*. The PCV2 genome contains at least five open reading frames (ORFs), of which the ORF2 encodes the Cap protein, which is involved in the assembly of the viral nucleocapsid, and is considered to be the most mutated and immunogenic viral protein [1,2]. PCV was first detected in a contaminated pig kidney cell line, PK-15, and named according to its characteristics [3]. In 1998, PCV variants were identified in piglets with progressive wasting disease [4]. Subsequently, non-pathogenic PCV derived from PK-15 cells was renamed PCV1, and the variant strain was called PCV2. Currently, PCVs are classified into four types: PCV1, PCV2, PCV3 [5], and PCV4 [6,7]. PCV2 and PCV3 are pathogenic to pigs and are distributed worldwide [8,9,10,11]. PCV2 is the main etiological agent causing PCVAD, as well as causing postweaning multisystemic wasting syndrome (PMWS), porcine dermatitis, and nephropathy syndrome (PDNS) [12,13,14,15], which ravages the swine industry worldwide, causing severe infection and huge economic losses [11,16,17,18]. More importantly, co-infection of PCV2 with other pig pathogens complicates disease management, and may benefit the proliferation of other pathogens or cause more serious clinical signs [19,20].

The estimated rate of nucleotide substitution for PCV2 is relatively high, making it comparable to RNA viruses, and is accompanied with recombination driving the development of new genotypes [21,22,23,24,25]. PCV2 can be divided into five genotypes: PCV2a, PCV2b, PCV2c, PCV2d, and PCV2e [22,26,27,28]. Some studies have also reported the existence of PCV2f [29,30], PCV2g [27,31], and PCV2h [32,33,34] genotypes. Some intermediate (IM) genotypes have also been proposed, revealing that recombination is also an important mechanism for the evolution of PCV2 [35,36]. PCV2e was discovered in 2015 in the United States and Mexico [37], albeit traced back to 2006 [15]. The nucleotide identity of the ORF2 gene is only 85%, which makes PCV2e a clearly new genotype. In addition, PCV2e sequences were identified in South Korea in 2019 [38], and were isolated in 2020 [39]. Therefore, continuous surveillance of emerging PCV2 genotypes is indispensable.

The prevalence of PCV2 in Chinese pig farms is high [33,40]. In 2017, the new PCV2e genotype was first detected in the Fujian province, southern China, indicating that there are still gaps in PCV2 diversity and prevalence due to its continuous evolution, thus stressing the necessity of continuous surveillance [41]. The purpose of this study was to investigate the prevalence and genetic variation of PCV2, especially PCV2e, as well as co-infection with other pathogens in some provinces of southern China from 2018 to 2021. This study provides a basis for the prevention and control of PCV2 in China.

## 2. Materials and Methods

### 2.1. Sample Collection and Preparation

A total of 79 lung samples from dead pigs with respiratory signs were collected from the Fujian, Guangdong, and Jiangxi provinces of southern China between 2018 and 2021. The lungs were cut into small pieces and homogenized in sterile phosphate-buffered saline (PBS). The supernatant was collected after centrifugation and stored at −80 °C. To isolate *S. suis*, the homogenized lung samples were cultured on Todd–Hewitt Broth (THB; OXOID, Basingstoke, UK) with 5% fetal bovine serum (THB + 5% FBS) on agar plates at 37 °C for 18 h, and the isolated *S. suis* were cultured in THB + 5% FBS broth at 37 °C for 18 h.

### 2.2. Nucleic Acid Extraction and Sequencing

A total 200 μL supernatant was subjected to RNA and DNA extraction, according to the manufacturer’s instructions, using EasyPure Viral DNA/RNA Kit (TransGen Biotech, Beijing, China). Reverse transcription was performed using the TransScript-Uni One-Step gDNA Removal and cDNA Synthesis SuperMix Kit (TransGen Biotech, Beijing, China). Bacterial DNA was extracted from culture broth using the bacterial genomic DNA kit. We used polymerase chain reaction (PCR) to detect common swine pathogens, including porcine reproductive and respiratory syndrome virus (PRRSV), porcine circovirus 3 (PCV3), classic swine fever virus (CSFV), pseudorabies virus (PRV) and *S. suis* [42,43]. The primers used for the detection of viral and bacterial genes are listed in Table S1. The genomes of PCV2-positive samples were amplified using two overlapping primer pairs (Table S1). The PCR procedure was as follows: 95 °C for 3 min, 35 cycles at 95 °C for 1 min, 56 °C for 1 min (different primer pairs choose different optimum temperatures), and 72 °C for 1 min, with a final extension at 72 °C for 10 min. The PCR product was sequenced by the Sanger method.

### 2.3. Sequence Alignment and Phylogenetic Reconstruction

Based on non-recombinant PCV2 sequences from a previous study [44], 104 reference sequences from the GenBank database were downloaded and analyzed, together with 23 newly sequenced whole-genome strains (accession number: MW974867-MW974889). Multiple sequence alignments of the nucleotide sequences of complete genomes and ORF2 were generated using the MUSCLE algorithm in MEGA7 [45], and adjusted manually. Maximum-likelihood (ML) trees were constructed with RAxML (version 8.2.12) [46], using the General Time-Reversible nucleotide substitution model with gamma distribution (GTR + Γ). Bootstrap analysis with 1000 replicates was used to determine the reliability of the generated trees. SSE V1.4 software was used to calculate pairwise distances (p-distance).

The PCV2 sequences from Fujian, Guangdong and Jiangxi provinces were downloaded from GenBank. After removing recombinant strains, Bayesian Markov Chain Monte Carlo (MCMC) analysis and population dynamics inference were performed using BEAST 1.10.4 [47]. The GTR + Г nucleotide substitution model was applied to the data set, under a strict molecular clock, with a prior Bayesian skyride for the tree topologies [48]. The MCMC runs included 2 × 10^7^ iterations, with parameter samples being taken every 2 × 10^3^ steps. The MCMC ran two independent replicates, and merged them using LogCombiner v1.10.4.

### 2.4. Selection Analysis

The Datamonkey web server (http://www.datamonkey.org, accessed on 10 March 2021) was used to infer the selection pressure on the ORF2 gene. We used four algorithms: SLAC (Single-Likelihood Ancestry Counting), FUBAR (Fast, Unconstrained Bayesian Approximation), MEME (Evolutionary Mixed Effects Model), and FEL (Fixed Impact Probability) to estimate the ratio of non-synonymous-to-synonymous substitution rate (dN/dS) and positively selected codons [49,50,51]. When the *p*-value was less than 0.1, the positive signal detected by SLAC, FEL and MEME was acceptable, while the posterior probability needed to be higher than 0.9 for FUBAR [1,52,53]. Positive selection sites were reported when the signal was observed by at least three algorithms.

## 3. Results

### 3.1. Prevalence and Co-Infection of PCV2 in Southern China

All 79 lung samples collected between 2018 and 2021 were identified by PCR, of which 23 were PCV2-positive. Table 1 describes the geographic origin, infection rate, and co-infection of PCV2 with other porcine pathogens. Among PCV2-positive samples, 69.6% (16/23) were co-infected with other porcine pathogens. Co-infection with CSFV and *S. suis* was the most frequent (26.1%), and was found in all three provinces. There was only one case of PCV2 co-infection with PRRSV, and two cases with PCV3. The co-infection rate with PRV was 21.7% (5/23). Most importantly, there were triple infections in Fujian: one case of PCV2, PRRSV, and *S. suis*, and two cases of PCV2, CSFV, and PRV. In the Guangdong province, co-infections of PCV2, PCV3, and *S. suis* were observed.

### 3.2. Phylogenetic Analysis and Population Dynamics Inference

**Figure 1:**
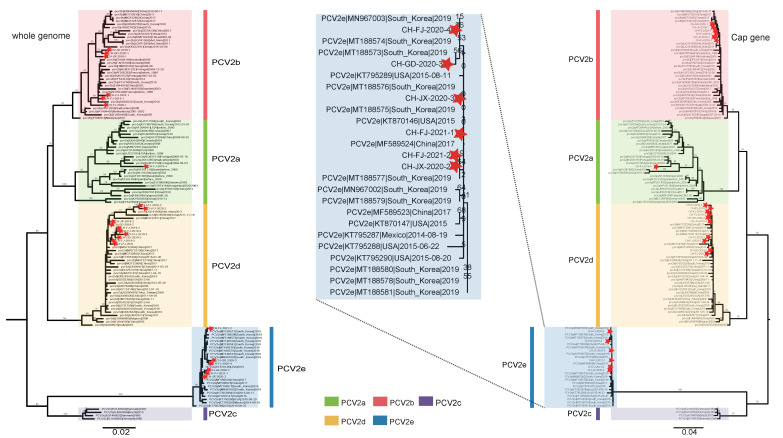
Maximum-likelihood (ML) trees of PCV2 whole genomes and the ORF2 gene. ML tree of PCV2 whole genomes. Scale Bar: 0.02 nucleotide substitutions per site (**left**). ML tree of the PCV2 ORF2 gene. Scale Bar: 0.04 nucleotide substitutions per site (**right**). The five-pointed stars represent the virus strains sequenced in this study. As in the prompt rectangle, different colors indicate different PCV2 genotypes.

**Figure 2:**
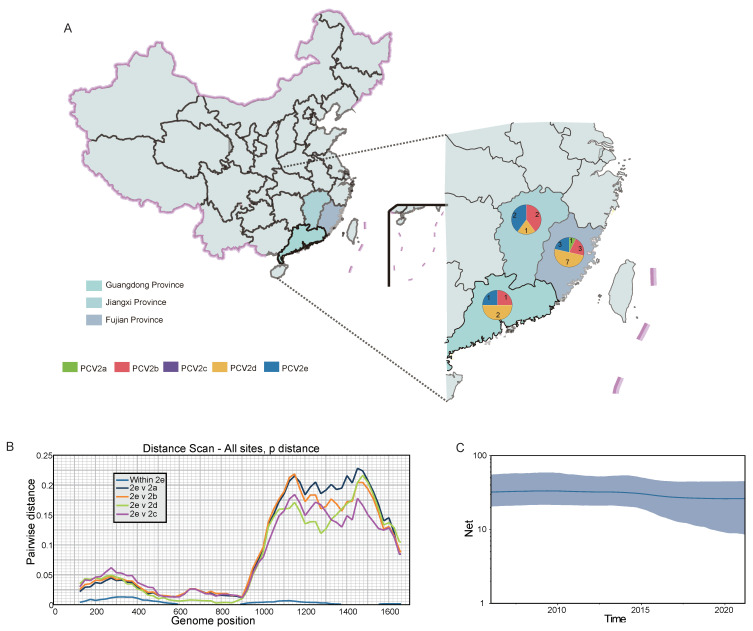
The distribution of sequenced samples, genetic distance, and the population dynamics of PCV2 in southern China. (**A**) Sampling sites. The pie chart and the number represent the proportion and number of PCV2 different genotypes. (**B**) P-distance within the PCV2e genotype and among other genotypes based on whole genomes. (**C**) Population dynamics of PCV2 in southern China. The PCV2 genotypes and provinces are color-coded in the rectangle.

All positive samples (*n* = 23) were successfully sequenced (accession numbers: MW974867-MW974889). Phylogenetic analysis was performed using the 23 PCV2 sequences from this study and 104 PCV2 reference sequences deposited in the GenBank database. Similar topologies were observed in the ML trees reconstructed based on the whole genomes and ORF2 genes. A total of five genotypes were identified: PCV2a, 2b, 2c, 2d, and 2e (Figure 1). The PCV2 strains sequenced here belong to four genotypes: PCV2a (1/23), PCV2b (6/23), PCV2d (10/23), and PCV2e (6/23). Genotypes PCV2b and PCV2d were detected in all provinces investigated, but PCV2a was found only in the Fujian province (Figure 2A). In the ML tree, the PCV2a strain from this study clustered with earlier PCV2a sequences from Switzerland and Portugal, and was close to the sequences in China in recent years, suggesting the latest PCV2a strains may retain the characteristics of the earlier PCV2a sequences with fewer variations. For the PCV2e clade, the strains in various countries were found to be scattered without any feature of geographic aggregation, owing to their high sequence similarity (Figure 1 and Figure 2B). Interestingly, PCV2e existed in all three provinces, and the Cap gene of PCV2e had low identity with other genotypes (Figure 2A,B). We found that the effective population size fluctuated slightly within the last 20 years, but overall remained at a stable level (Figure 2C).

### 3.3. ORF2 Amino Acid Analysis

**Figure 3:**
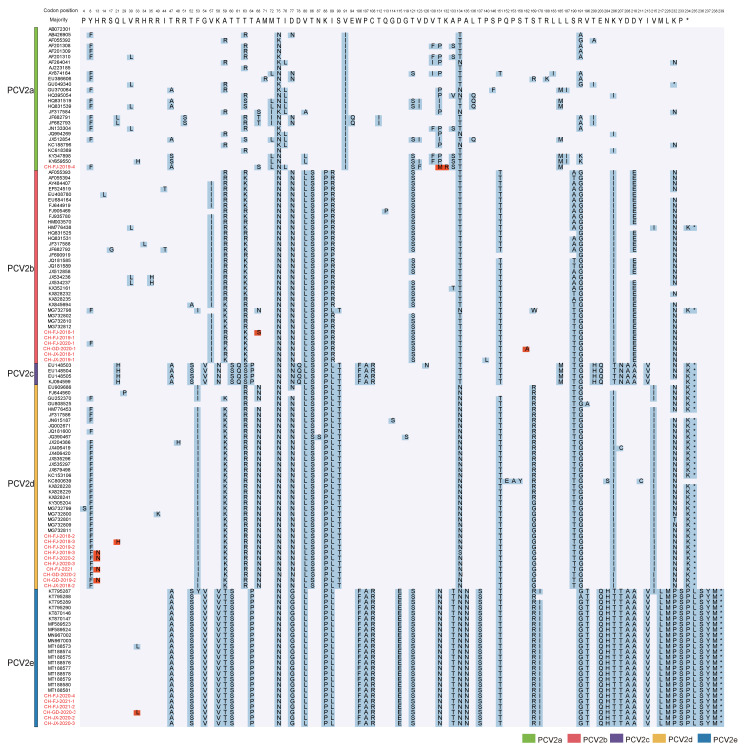
Analysis of amino acid variation of the PCV2 Cap protein. Pairwise alignment of the amino acid of 104 PCV2 cap protein reference strains and 23 sequenced strains. The residues that are the same as the first reference sequence are not marked; only the different residues are listed and distinguished by different background colors. The strains sequenced here, and the variants only found in this study, are highlighted in red.

We performed pairwise amino acid sequence comparisons to understand the homogeneity of the protein encoded by ORF2. Figure 3 depicts the mutation sites in the PCV2 reference sequences and the 23 PCV2 sequences from this study, highlighting the highly variable regions (mainly located in amino acid positions 57–91, 121–151, and 190–210) [52,53] and some specific mutation sites that can distinguish genotypes [41]. For example, PCV2a can be distinguished from other genotypes by the amino acid in position 91, which is Ile for PCV2a and Val for the rest of the PCV2 genotypes. Similarly, there are unique mutations in PCV2b and PCV2d, namely Ile^57^, Arg^89^/Pro^89^, Glu^210^ on PCV2b, and Ile^53^, Asn^68^, and Ile^215^ on PCV2d. Of note, PCV2c and PCV2e have low identity with the other three genotypes based on the ORF2 gene but share some amino acid variations with each other (Ser^52^, Val^54^, Ser^60^, Pro^64^, Phe^106^, Ala^107^, Arg^108^, Gln^203^, Thr^206^, Ala^208^, Ala^210^, Val^213^). For PCV2e, the 12-nucleotide insertion at the end promotes the uniqueness of amino acid positions 232 to 238. In addition, some scattered unique mutations also exist on PCV2e (Val^58^, Thr^59^, Gly^77^, Glu^115^, Asn^131^, Thr^133^, Asn^135^, Ser^137^, Ile^170^, Thr^196^, His^204^, His^207^, Leu^217^, Met^226^) [41]. The PCV2e strains sequenced here are conserved despite a variation from Arg to Leu at position 33 on the CH-GD-2020-3 strain. Some strains sequenced here have unique mutations. For example, codons 131 and 132 of the CH-FJ-2019-4 strain are Met and Arg, respectively. The CH-FJ-2018-1 strain mutated from Ala to Ser at codon 68, which is rare in PCV2b. The CH-GD-2020-1 strain changed from Thr to Ala at position 162. For the CH-FJ-2018-3 strain, position 21 has the same amino acid as the PCV2c genotype, which mutated from Gln to His. Interestingly, 4 of the 10 sequenced PCV2d strains have unique mutations at position 13 (from His to Asn). Amino acid analysis confirmed the existence of PCV2e in southern China, and there is no significant difference in PCV2e strains.

### 3.4. Selection Analysis

Two sites at position 63 and 133 in the ORF2 were positively selected sites, as detected by more than three methods (Table 2), in agreement with previous studies [1,52,53,54]. Moreover, the two identified positive-selection sites may be involved in the formation of PCV2 epitopes [55,56]. Of note, even using stricter criteria (for SLAC, FEL and MEME, *p*-value < 0.05, and posterior probability of FUBAR > 0.95), codons at position 63 still displayed selection signals with diverse amino acids at this position: mainly Thr, Arg, Ser on PCV2a; Arg, Lys on PCV2b; Ser on PCV2c; Arg on PCV2d; and Thr on PCV2e.

## 4. Discussion

PCV2 is a ubiquitous viral pathogen that causes PCVAD in pigs [54,57]. Relatively high mutation and recombination rates drive increased genetic diversity and the emergence of new genotypes in PCV2, which can even lead to dominant genotype shifts [58], such as the changes from the dominant genotype PCV2a to PCV2b in 2003 [18,59], and now PCV2b being replaced by PCV2d [28,59,60,61]. The distribution and genetic features of PCV2a, PCV2b, and PCV2d genotypes have been extensively reported worldwide, while little is known about PCV2c and emerging PCV2e genotypes. Recently, the newly emerging PCV2e was reported by more countries, and isolated from Korean pigs for the first time. PCV2e with high heterogeneous Cap gene increases the possibility of the next genotype shift. Interestingly, the current commercial PCV2 vaccines commonly used in pig farms are based on the PCV2a genotype, and it is unknown if they are protective against PCV2e. In view of the high mutation rate of PCV2 and the emergence of the PCV2e genotype, routine surveillance of PCV2 is essential. Here, we used a non-recombinant sequence data set to explore the variation of PCV2 in southern China in the past three years.

In this study, PCV2 positivity was 29.1%, and co-infections of PCV2 with multiple porcine-derived pathogens were frequent. Notably, mixed infection can exacerbate PCV2 pathogenicity [62]. Previous reports have shown that co-infection of different viruses and bacteria usually synergistically exacerbates the pathogenicity of microorganisms [62], causing high morbidity and mortality in animals [63,64]. For example, simultaneous infection of *S. suis* with PRRSV and swine influenza virus promoted more serious respiratory disease [65,66]. Another study reported that co-infection between PCV2 and *S. suis* resulted in more serious tissue lesions and enhanced pathogenicity in piglets [67]. The impact of mixed infections makes it impossible to ignore the research on PCV2. Interestingly, only one case of PCV2 co-infected with PRRSV here, while previous reports reveal that PRRSV and PCV2 are always found together [28,67,68,69]. This may be a bias caused by our small sample size and only-lung sample type, but also indicates another possibility: an unknown new pathogen causing pig death. Notably, it is common for new or recurrent pathogens with unknown origins to cause outbreaks in Chinese pig herds [70,71,72], which suggests that strengthening pathogens’ surveillance in pig herds is extremely important for both the pig industry and public health safety [72].

Based on ML trees of whole genomes and ORF2 genes, we identified for the first time the presence of PCV2e in the three investigated provinces. We confirmed the high prevalence of PCV2b and PCV2d and the low prevalence of PCV2a, which is consistent with other studies [33,64,71,72]. Notably, due to the use of non-recombinant sequence sets, the five genotypes are well-defined. However, in fact, given that the diversity of PCV2 is high and the PCV2 evolution and recombination mechanisms are complicated, there is the constant appearance of new genotypes, such as PCV2f and PCV2g [27,29]. Knowing that different PCV2 genotypes are prevalent in China, and the newly emerged PCV2e genotype is present in southern China, we performed a deeper amino acid analysis of the ORF2 gene. We found some amino acid changes at epitopes in the Cap protein. In particular, codons at position 13 (His) of the Cap protein of the four PCV2d strains from this study were Asn, indicating adaptive mutation and the need for further analysis. In the selection analysis, we found two positively selected sites, which are related to antigenic epitopes. Interestingly, codons at position 63 are under strong selection pressure and varied in genotypes, especially for PCV2c, PCV2d, and PCV2e, which seems to be fixed as Ser, Arg and Thr, respectively, indicating that natural selection may have a screening effect on the PCV2 genotype.

Vaccines are an important means of preventing epidemics. Currently, available commercial PCV2 vaccines based on the PCV2a genotype are highly effective, and provide cross-protection against PCV2b and PCV2d [73,74]. Meanwhile, vaccine pressure may drive the evolution of PCV2 [75]. For PCV2e, there are significant differences between the amino acids of the Cap protein and other genotypes; as such, whether the available vaccines could protect against PCV2e is still unknown. Therefore, it is necessary to investigate the prevalence of PCV2e in China and to reassess the protective effect of the available vaccines.

In conclusion, this study reveals that PCV2e is an emerging genotype circulating in southern China. Considering the significant contributions of swine farming to the regional livestock economy, and increasing demand for local pork in southern China, our findings emphasize the importance of surveillance of emerging PCV2e and genotyping of PCV2 in this region, to help develop strategies for the prevention and control of PCV2e.

## Figures and Tables

**Table 1:** Co-infection of PCV2-positive samples with other porcine-associated pathogens.

	Fujian	Guangdong	Jiangxi	Sum	
Positive number	14	4	5	23	
Co-infection positives	11	2	3	16	
	number of infections			number of infections	co-infection rate
Double infection					
PCV3	1	0	0	1	4.35%
CSFV	2	1	1	4	17.39%
PRV	2	0	1	3	13.04%
PRRSV	0	0	0	0	0
*S. suis*	3	0	1	4	17.39%
Triple infection					
*S. suis* + PRRSV	1	0	0	1	4.35%
*S. suis* + PCV3	0	1	0	1	4.35%
CSFV + PRV	2	0	0	2	8.70%
The total number of co-infections with a certain pathogen					
PCV3	1	1	0	2	8.70%
CSFV	4	1	1	6	26.09%
PRV	4	0	1	5	21.74%
PRRSV	1	0	0	1	4.35%
*S. suis*	4	1	1	6	26.09%

**Table 2:** Selection analysis of PCV2 ORF2 gene.

Codon Position	*p*-Value			Posterior Probability
	FEL	MEME	FEL	FUBAR
63	0.002	0.010	0.0316	0.985
133	0.090	0.080	0.0878	0.936

## Data Availability

All data are available in the main text or the Supplementary Materials. The sequences data presented here are openly available in GenBank: MW974867-MW974889.

## Supplementary Materials

The following supporting information can be downloaded at: https://www.mdpi.com/article/10.3390/v14040724/s1, Table S1: Specific primers used in this study [42,76,77,78].
