# Supplementary material for: Epidemiology and Genetic Diversity of PCV2 Reveals That PCV2e Is an Emerging Genotype in Southern China: A Preliminary Study
Source: Viruses. 2022 Mar 30;14(4):724. doi: 10.3390/v14040724 (PMC9026887; doi:10.3390/v14040724)
Supplement: Supplementary file 1 [file viruses-14-00724-s001.zip › viruses-1618959-supplementary.pdf]

Supplementary Table S1. Specific primers used in this study

Primer Name	Sequence (5'-3')	PCR product size (bp)	References
S. suis			
16S rRNA-F	CAGTATTTACCGCATGGTAGATAT	319 (Detection of 16S rRNA)	[42]
16S rRNA-R	GTAAGATACCGTCAAGTGAGAA		
recN-F	CTACAAACAGCTCTCTTCT	336 (Detection of recN)	
recN-R	ACAACAGCCAATTCATGGCGTGATT		
PCV2			
PCV2-D-F	AGAAGCTCTCTATCGGAG	569	[76]
PCV2-D-R	AAGGTTGAATTCTGGCCC		
PCV2-C-F1	TGGTGACCGTTGCAGAGCAG	1103	
PCV2-C-R1	TGGGCGGTGGACATGATGAG		
PCV2-C-F2	KGTATGGCGGGAGGAGTAG	1123	
PCV2-C-R2	AGGTGGTTTCCAGTATGTGG		
PCV3			
PCV3-D-F	TTACTTAGAGAACGGACTTGTAACG	649	[77]
PCV3-D-R	AAATGAGACACAGAGCTATATTCAG		
CSFV			
CSFV-D-F	TAGGGTGGACGGGTGTCATAGAGT	566	[76]
CSFV-D-R	AAGCATATATTGCTGGAAGTAGCT		
PRRSV			
PRRSV-D-F	GCCTCGTGTTGGGTGGCAGAA	532	
PRRSV-D-R	CGCCCTAATTGAATAGGTGACTT		
PRV			
PRV-D-F	GAAGGGGTTGGACAGGAAGGAC	194	[78]
PRV-D-R	ACCAGCCGCTCAAGTTCTACGA		
